# Impact of Atrazine on Sucrose Sensitivity in Honey Bees

**DOI:** 10.3390/insects16050491

**Published:** 2025-05-03

**Authors:** Xiexin Hu, Zixuan Xu, Jiachen Xu, Guiyi Ma, Yiren Pan, Minqi Cai, Zheguang Lin, Ting Ji, Kang Wang

**Affiliations:** 1 College of Bioscience and Biotechnology, Yangzhou University, Yangzhou 225009, China; huxiexin@outlook.com; 2 College of Animal Science and Technology, Yangzhou University, Yangzhou 225009, China

**Keywords:** honey bees, atrazine, sucrose sensitivity, neurotoxicity, RNA sequencing

## Abstract

Honey bees (*Apis mellifera*) are essential pollinators, responsible for the pollination of over 80% of crops and flowering plants globally. However, there is concern that the extensive use of pesticides, particularly atrazine, can harm pollinators. Despite the widespread use of atrazine, the sublethal effects on honey bees remain unclear. This study investigated the effects of atrazine on honey bee sucrose sensitivity and clarified the underlying molecular mechanisms using transcriptomic analyses. Atrazine exposure reduced the sucrose sensitivity of honey bees substantially, likely through the inhibition of functional genes associated with cognition in the brain. Genes related to neurodegenerative diseases and behavior were differentially expressed in response to atrazine. These findings provide novel insights into the neurophysiological and behavioral effects of atrazine on honey bees, contributing to a better understanding of pesticide risks and informing future environmental regulations.

## Simple Summary

Honey bees play a crucial role in pollinating crops and maintaining biodiversity; however, their populations are declining due to various environmental threats, including pesticide exposure. In this study, we investigated the effects of atrazine, a widely used herbicide, on honey bee behavior and brain function. Specifically, we tested whether atrazine exposure influences the ability of bees to sense sweetness, which is essential for finding food. Our results showed that bees exposed to atrazine had a reduced ability to detect sucrose solution, potentially impairing their foraging efficiency. A transcriptomic analysis revealed significant changes in gene expression levels in the brain, particularly in genes linked to nerve function and memory. These findings suggest that even low doses of atrazine can negatively impact honey bee cognition, which may contribute to broader declines in bee populations. Understanding these effects is critical for developing safer agricultural practices and protecting pollinators, which are vital for food production and ecosystem stability. Our research highlights the need for stricter regulations on pesticide use to ensure the health of bees and the sustainability of global agriculture.

## 1. Introduction

Honey bees (*Apis mellifera*) are the most important pollinators in nature and are responsible for pollinating over 80% of crops and flowering plants globally [1]. Since the 20th century, the widespread use of pesticides has led to mass bee deaths and a drastic reduction in bee populations across many countries [2,3]. This has become a globally recognized issue in terms of environmental pollution and biological safety. Over the past decade, the ban on high-toxicity pesticides has led to the increased use of low- and moderate-toxicity pesticides. Although deaths due to acute poisoning in bee populations have become less frequent, the sublethal effect of low-toxicity pesticides on bees has attracted increasing attention from researchers [4].

Atrazine, the second most widely used herbicide globally, has raised considerable concerns owing to its pervasive and improper application, resulting in severe environmental contamination and associated health risks [5]. It exhibits a broad spectrum of toxicological effects across various animal taxa, functioning as a neurotoxin, reproductive toxin, and endocrine disruptor [6]. There is increasing evidence that atrazine exerts detrimental effects on insect species. For instance, atrazine exposure disrupts immune system regulation in honey bees, thereby increasing their susceptibility to pathogenic infections [7]. In addition, experimental studies have demonstrated that atrazine exposure adversely affects longevity, prolongs developmental duration, and reduces body size in *Drosophila melanogaster* [8]. Female flies exposed to atrazine show a reduction in exploratory activity, an increase in immobility time, and dopamine system disruption [9]. Moreover, adult insects have been reported to exhibit a greater sensitivity to atrazine and selenium compared with that at larval stages, and the deleterious effects of these contaminants are more pronounced in terrestrial ecosystems than in aquatic environments [10]. Atrazine demonstrates significant neurotoxic properties in vertebrate models. Experimental studies in zebrafish *(Danio rerio)* have established that this herbicide induces behavioral deficits in both defensive and social responses through the specific inhibition of acetylcholinesterase activity in central nervous system tissues [11]. The hippocampus, a critical brain region responsible for learning and memory, is particularly susceptible to such effects in mice (*Mus musculus*) [12,13]. Exposure to atrazine during pregnancy and lactation in mice leads to increased apoptosis and dark neurons in the hippocampus of offspring, ultimately impairing their learning and spatial memory abilities [14]. Genovese et al. [15] found that atrazine exposure leads to behavioral changes and impaired motor memory in mice, with more pronounced effects in old mice than in young mice.

Research on the sublethal effects of atrazine on honey bees remains limited [5], despite the widespread use of this agent and the importance of honey bees for food production and ecosystem stability. In this study, we evaluated the impact of atrazine on honey bee’s sucrose sensitivity and explored the underlying mechanisms through a transcriptomic analysis. By elucidating the effects of atrazine on honey bees at the molecular and behavioral levels, this research contributes to a deeper understanding of pesticide-induced stress in pollinators and provides a basis for conservation strategies and regulatory policies to mitigate the risks posed by herbicide exposure.

## 2. Materials and Methods

### 2.1. Honey Bees and Atrazine Preparation

Honey bees (*Apis mellifera*) were raised at Yangzhou University, China. Atrazine (99.17% TC; MedChemExpress, Princeton, NJ, USA) powder was initially dissolved in dimethyl sulfoxide, and 0.5% (3.73 mg/L) of the median lethal dose (LD_50_ = 746 mg/kg) for honey bees was used as the test dose [16]. Exposure to atrazine for six consecutive days did not lead to any observable increase in honey bee mortality under the tested conditions in our previous study [7]. 

### 2.2. Proboscis Extension Response (PER) Experiment 

Three frames from different hives were placed in a dark incubator at 34 °C and 70% relative humidity. Newly emerged worker bees from different frames were divided into two groups, with three cup cages per group as biological replicates, each consisting of 20–30 bees. All bees were fed ample pollen bread and sucrose solution containing either 0 mg/L (CK) or 3.73 mg/L atrazine (AT) for 6 d [7]. 

Individual gustatory responses were measured using the classical proboscis extension response (PER) experiment [17]. The PER is a taste related behavior that is fundamental for olfactory discrimination and colony performance in honey bees. Six-day-old bees were tested for their proboscis extension response to sucrose solutions at concentrations of 0.1%, 0.3%, 1%, 3%, 10%, and 30%, following previously established protocols [18], with some modifications. Before the test, the bees were starved for 3–4 h in an incubator by removing the sucrose solution and bee bread from the cup cage. All bees were anesthetized by chilling and fixed in small tubes with only their heads moving. Before the presentation of each sucrose solution, bees that reacted to pure water or did not react to high concentrations of sucrose solution were excluded. Honey bees will reflexively extend the proboscis when the antennae are stimulated by a sufficiently concentrated sucrose solution. To weaken the effect of physical stress, the stimulation interval between water and sucrose solution was 5 min. The response was arbitrarily quantified with scores of 0–6, with 1 representing a response only to the highest sucrose concentration and 6 representing a response to all concentrations presented (Table S1). After the PER experiment, all bee brains were dissected and stored in a −80 °C ultra-low-temperature environment. 

### 2.3. Brain Gene Expression Analysis

Gene expression profiles in the brains of the bees were evaluated. Each group contained three biological replicates. RNA was extracted from honey bee brains according to the manufacturer’s recommendations (Vazyme, Nanjing, China) and sequenced using the Illumina NovaSeq 6000 Sequencing System. Raw reads containing adapters or low-quality bases were filtered using fastp 0.18.0. Clean reads were mapped to the *A. mellifera* reference genome, HAv3.1 (GenBank accession number GCA_003254395.2), using HISAT2 2.1.0. Subsequently, a gene count table was obtained using StringTie, and differentially expressed genes (DEGs) were identified using DESeq2 (version 1.32.0). Genes with adjusted *p* < 0.05 and |fold-change| ≥ 1.5 were considered significantly differentially expressed. 

A hierarchical clustering analysis of DEGs was performed to examine global expression patterns. Gene Ontology (GO) enrichment analysis was subsequently conducted to classify DEGs into functionally relevant categories, including biological processes, molecular functions, and cellular components. Additionally, Kyoto Encyclopedia of Genes and Genomes (KEGG) pathway enrichment analysis was carried out using a hypergeometric distribution model to identify significantly overrepresented metabolic and signaling pathways. Functional annotation and pathway mapping of DEGs were conducted based on the KEGG database to gain insights into the underlying biological mechanisms.

To validate the differential gene expression identified through RNA sequencing, quantitative reverse transcription PCR (qRT-PCR) was performed using a QuantStudio™ 3 Real-Time PCR System (Thermo Fisher Scientific, Waltham, MA, USA). Total RNA was extracted as previously described [7] and subsequently reverse-transcribed into complementary DNA using a commercial kit (Vazyme, Nanjing, China). The synthesized cDNA was then used as a template for quantitative analysis of selected genes following the manufacturer’s qPCR protocol (Vazyme, Nanjing, China). Relative gene expression levels between control and atrazine-treated groups were calculated using the 2^−ΔΔCT^ method, with *Apis mellifera* actin employed as the endogenous reference gene. Gene-specific primers were designed using NCBI Primer-BLAST and are listed in Supplementary Table S2.

### 2.4. Statistical Analysis

Sucrose sensitivity scores for the two groups of bees were analyzed using Mann–Whitney U tests (* *p* < 0.05; ** *p* < 0.01; *** *p* < 0.001) implemented in SPSS 27.0 software. Bioinformatic analyses of gene expression and visualization were performed using R software.

## 3. Results

### 3.1. Atrazine Reduces Sucrose Sensitivity in Honey Bees

**Figure 1:**
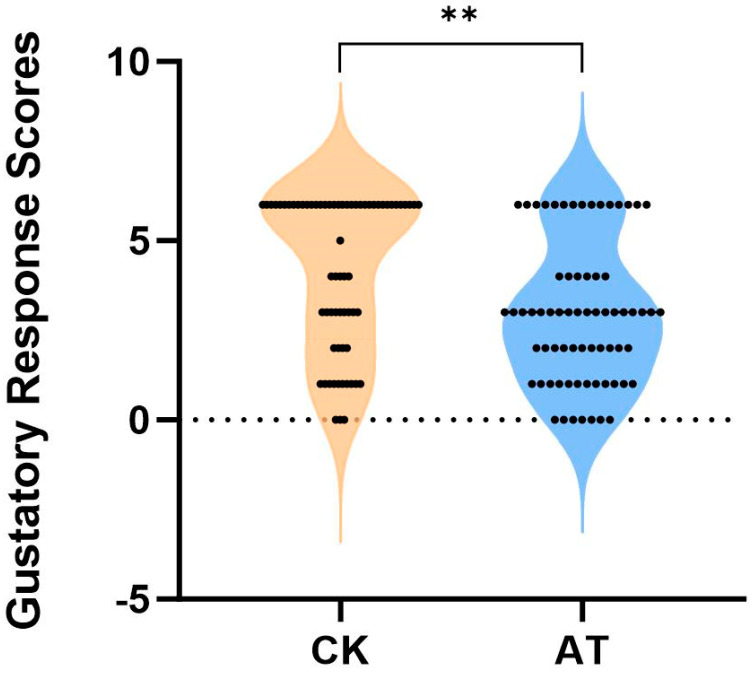
Comparison of sucrose sensitivity with and without atrazine treatment in the honey bee. CK: normal honey bee (*n* = 68); AT: atrazine-treated honey bee (*n* = 71); atrazine reduced honey bee sensitivity to sucrose significantly. Mann Whitney U test, ** *p* < 0.01.

The gustatory response scores for honey bees exposed to atrazine were significantly lower than those in the control group (*p* < 0.01); the scores in the atrazine group were 70.92% of those in the control group (Figure 1). These results suggested that atrazine exposure impairs the sucrose sensitivity of bees.

### 3.2. Atrazine Changes the Functional Gene Expression in Honey Bee Brain

**Figure 2:**
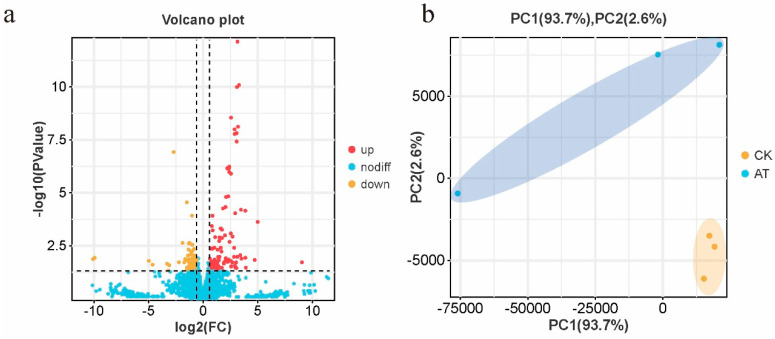
Transcriptome profiles of the brains of atrazine-treated and normal honey bees. (**a**) In total, 10,448 genes were detected through RNA sequencing, including 112 significantly upregulated (red) and 72 downregulated (yellow) genes in atrazine-treated honey bee brains compared with normal honey bees. (**b**) Principal coordinate analysis of the gene expression profiles. Samples in the control group were clustered, whereas samples in the atrazine-treated group were more dispersed.

The differential gene expression patterns following the atrazine treatment were analyzed to elucidate the molecular mechanisms underlying the altered olfactory sensitivities in honey bees. In total, 10,448 DEGs were identified (Table S3). Among these, 112 genes were upregulated and 72 genes were downregulated in the brains of atrazine-treated honey bees (Figure 2a; Table S3), indicating a significant shift in the gene expression associated with atrazine exposure. The control group showed less variation in gene expression than that in atrazine-treated bees (Figure 2b), suggesting variability in the gene expression responses among individuals exposed to the chemical stressor.

Detoxification pathways play a crucial role in the honey bee response to xenobiotic stress, and our findings indicate that these pathways were activated in response to atrazine exposure. Specifically, detoxification related genes, such as cytochrome P450 9e2, 4C1, and glutathione S-transferase D1, were significantly upregulated, which is consistent with the activation of the detoxification system. However, the expression of several functional genes, particularly those related to neuronal function, was suppressed in honey bees exposed to atrazine. For example, Neprilysin-2, FoxP, ataxin-2, and the neuropeptide Y receptor were downregulated. 

**Figure 3:**
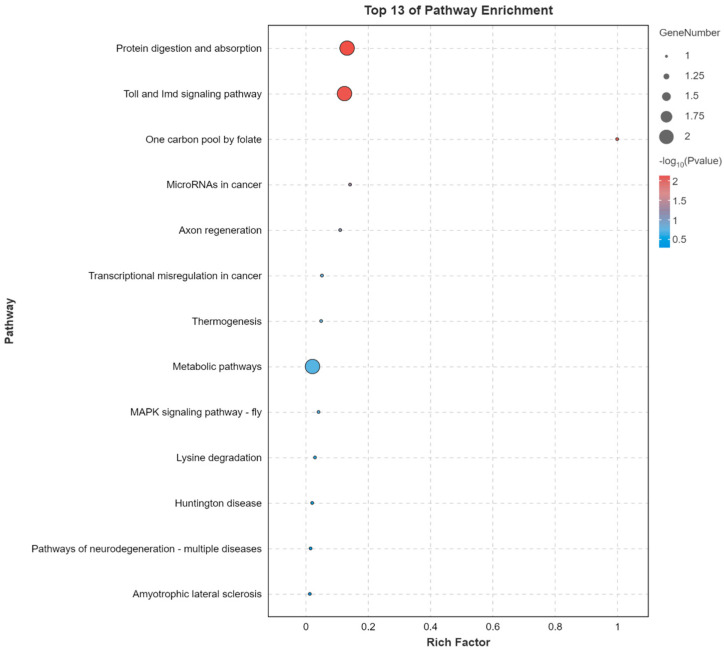
Top 13 significantly enriched Kyoto Encyclopedia of Genes and Genomes (KEGG) pathways associated with differentially expressed genes (DEGs). The *x*-axis indicates the KEGG enrichment score and the *y*-axis indicates the pathway names. Rich factor is a measure of the enrichment of a given pathway, calculated as the ratio of the number of DEGs in a pathway to the total number of genes involved in that pathway.

To gain insights into the functional implications of the DEGs, a GO enrichment analysis focusing on the molecular function domain, along with a KEGG pathway enrichment analysis, was performed. In the molecular function category of the GO analysis, DEGs were primarily enriched in terms such as the binding and catalytic activity, followed by the transporter activity and structural molecule activity (Figure S1). These results suggest that the identified DEGs are predominantly involved in molecular interactions, enzymatic activities, and transport related functions. The KEGG pathway enrichment analysis further revealed several significantly enriched pathways (Figure 3). Among these, protein digestion and absorption, and the Toll and Imd signaling pathway were the most prominent, indicating potential disruptions in nutrient utilization and innate immune responses. Notably, pathway associated with multiple neurodegenerative disease was enriched. These results suggest that atrazine exposure may interfere with neural development, which could contribute to the observed reduction in sucrose sensitivity.

The genes selected for validation were primarily involved in neural function. The RT-qPCR analysis confirmed their significant downregulation in atrazine-treated bees, in agreement with the RNA-seq results (Figure S2). This transcriptional repression suggests that atrazine exposure may disrupt neuronal processes, potentially contributing to the behavioral deficits observed.

## 4. Discussion

Atrazine is the second most widely used pesticide in commercial agriculture worldwide [19]. However, its effects on honey bee neurons have not been well studied. The current study aimed to evaluate the sublethal effects of atrazine on honey bee sucrose sensitivity and elucidate the potential underlying molecular mechanisms using a transcriptomic approach. The results provide strong evidence that atrazine exposure alters the sensory and behavioral responses of honey bees substantially, specifically their sucrose sensitivity. 

Olfaction plays a crucial role in various aspects of the honey bee ecology, including social communication, task division, queen rearing, individual development, swarming, and reproduction [20]. Honey bees demonstrate flower constancy because of their advanced sensory abilities and efficient capacity to learn and remember specific odors, which increases the ability of a colony to forage in a precise and efficient manner [21].

Previous studies have shown that exposure to pesticides, such as glyphosate, can alter honey bee preferences for certain food sources, which may further disrupt the foraging efficiency and colony health. These changes in preference, combined with a reduced sucrose sensitivity, could have significant implications for honey bee behavior and their role in pollination [22,23]. In this study, atrazine-treated honey bees exhibited a marked reduction in sucrose sensitivity, as measured by the PER, suggesting that atrazine impairs the ability of bees to effectively detect and respond to feed sources. This, in turn, could directly affect their foraging behavior and, consequently, their role in pollination.

Detoxification pathways play important roles in the ability of honey bees to cope with xenobiotic stress [24]. In general, the toxin structure is enzymatically altered primarily by the cytochrome P450 monooxygenase gene family and then solubilized and transported by detoxification enzymes, such as glutathione *S*-transferases [24]. The expression levels of some genes related to detoxification, such as cytochrome P450 9e2, 4C1, and glutathione *S*-transferase D1, were elevated, suggesting that the detoxification system of bees was activated. Glutathione *S*-transferase may be upregulated to reduce atrazine-induced oxidative stress [25,26]. Genes related to the Toll and Imd signaling pathways, which are crucial for immune regulation, were also upregulated in the atrazine-treated honey bees. This suggests that atrazine exposure activates immune related processes, potentially affecting the ability to respond to environmental stressors. Low-dose atrazine exposure induces DNA damage and activates glutathione S-transferase activity in zebrafish. However, this enzymatic activity shows a concentration-dependent inhibition at higher atrazine concentrations, suggesting the compromised detoxification capacity of the antioxidant defense system [27].

Various functional genes were suppressed when honey bees were exposed to atrazine, including neuron related genes. Neprilysin-2, a protease that degrades the amyloid-β peptide [27], has been shown to play an important role in protecting against Alzheimer’s disease [28,29,30,31,32] and is positively associated with cognitive function in humans. Although these findings are based on mammalian studies, they provide valuable insights that may offer a reference point for understanding the potential neural functions of Neprilysin-2 in honey bees, despite the phylogenetic distance between species. *Drosophila* FoxP plays an important role in α-lobe mushroom body formation and is believed to be involved in neurodevelopmental processes and behaviors; individuals with mutant FoxP show mild cognitive impairment [33,34]. In addition to Neprilysin-2 and FoxP, ataxin-2-, neuropeptide Y receptor-, and neurofilament heavy polypeptide-like proteins function in various neurodegenerative diseases [35,36]. Moreover, KEGG pathway analysis indicated that several DEGs in honey bee brains were associated with neurodegenerative disease related signaling pathways (Figure 3). The observed downregulation of these functional genes suggests a potential disruption of neural processes following atrazine exposure, which may underlie the reduced sucrose sensitivity observed in treated bees.

## 5. Conclusions

In conclusion, this study provides preliminary evidence that sublethal atrazine exposure can modulate sucrose sensitivity and alter the brain gene expression in honey bees under laboratory conditions. These findings highlight the potential risk that even low-dose pesticide exposure may pose to pollinator health. However, since the current study was conducted under controlled laboratory conditions, further comprehensive evaluations under field conditions are necessary to fully assess the ecological relevance and broader impacts of atrazine exposure.

## Data Availability

Raw sequence reads have been deposited in the NCBI SRA database under the BioProject accession no. PRJNA783076.

## Supplementary Materials

The following supporting information can be downloaded at: https://www.mdpi.com/article/10.3390/insects16050491/s1, Figure S1: GO enrichment analysis of DEGs in the molecular function category; Figure S2: Confirmation of DEGs by RT-qPCR. Table S1: Scoring Methods of PER; Table S2: Primers used in this study; Table S3: Gene expression analysis.

## Abbreviations

The following abbreviations are used in this manuscript:

PER	Proboscis extension response
DEG	Differentially expressed gene
KEGG	Kyoto Encyclopedia of Genes and Genomes
