# Supplementary material for: Impact of Atrazine on Sucrose Sensitivity in Honey Bees
Source: Insects. 2025 May 3;16(5):491. doi: 10.3390/insects16050491 (PMC12112258; doi:10.3390/insects16050491)
Supplement: Supplementary file 1 [file insects-16-00491-s001.zip › Figure S1.pdf]

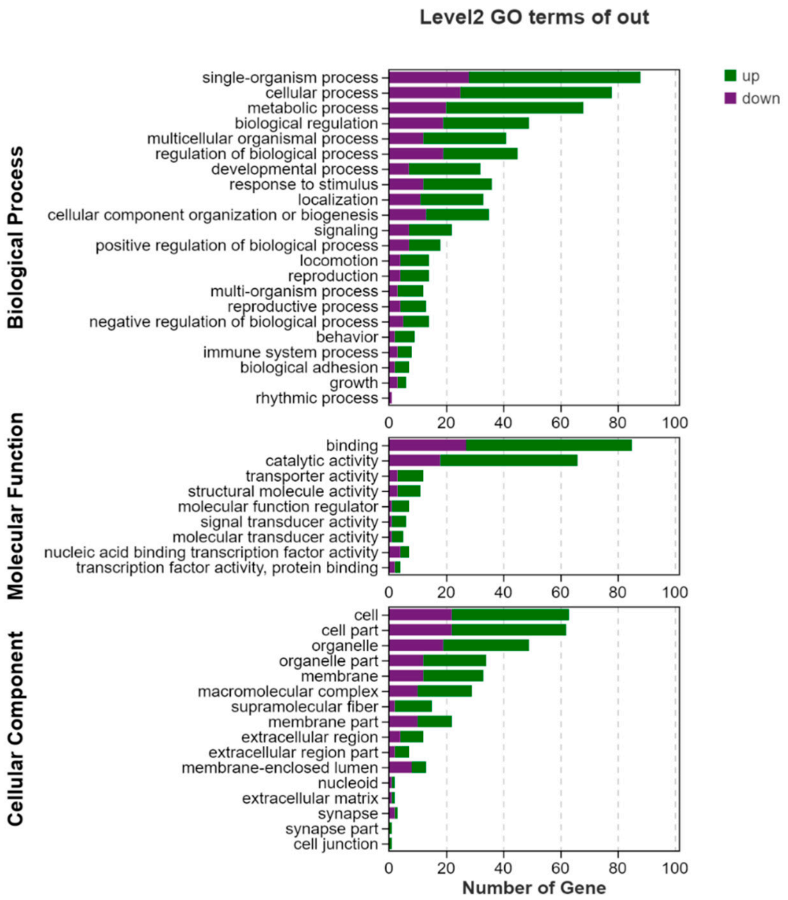

Figure S1. GO enrichment analysis of DEGs in the molecular function category. Green and purple indicate up- and downregulated genes, respectively.
