# Supplementary material for: Impact of Atrazine on Sucrose Sensitivity in Honey Bees
Source: Insects. 2025 May 3;16(5):491. doi: 10.3390/insects16050491 (PMC12112258; doi:10.3390/insects16050491)
Supplement: Supplementary file 1 [file insects-16-00491-s001.zip › Figure S2.pdf]

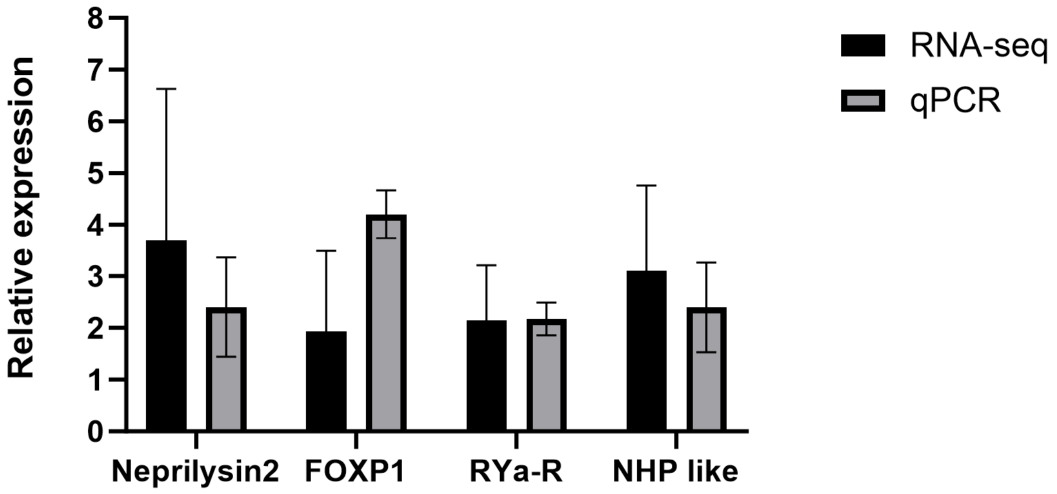

Figure S2. Confirmation of DEGs by RT-qPCR. Normalized gene expression levels are presented as the ratio of control to atrazine-treated bees for each gene, based on both RNA-seq and qPCR results.
