# Supplementary material for: Impact of Atrazine on Sucrose Sensitivity in Honey Bees
Source: Insects. 2025 May 3;16(5):491. doi: 10.3390/insects16050491 (PMC12112258; doi:10.3390/insects16050491)
Supplement: Supplementary file 1 [file insects-16-00491-s001.zip › Table S1.pdf]

Table S1 Scoring Methods of PER

Sugar water concentration	0.1%	0.3%	1%	3%	10%	30%	PER score
	1	1	1	1	1	1	6
	0	1	1	1	1	1	5
	0	0	1	1	1	1	4
score	0	0	0	1	1	1	3
	0	0	0	0	1	1	2
	0	0	0	0	0	1	1
	0	0	0	0	0	0	0
