# Supplementary material for: Impact of Atrazine on Sucrose Sensitivity in Honey Bees
Source: Insects. 2025 May 3;16(5):491. doi: 10.3390/insects16050491 (PMC12112258; doi:10.3390/insects16050491)
Supplement: Supplementary file 1 [file insects-16-00491-s001.zip › Table S2.pdf]

Table S2 Primers used in this study.

Target	Direction	Sequence
abaecin	forward	TCGGATTGAATGGTCCCTGAC
	reverse	ATCTTCGCACTACTCGCCAC
Neprilysin2	forward	TCGGTCACGAGTTGACTCAC
	reverse	TCCGTCTACCTGTTCAGCTT
FOXP1	forward	CAATCTCCAGGCGGCACT
	reverse	TATGGGCTAACACGTGCTC
RYa-R	forward	TCAACATCGTGGCAAGATCA
	reverse	GGACCATCCAACGAGCCATT
NHP like	forward	GAAGCGTCGGCAGAGAAGAA
	reverse	CGGGTAACGTGATGGCTGAA
